# Supplementary material for: Co-transcriptional R-loops are the main cause of estrogen-induced DNA damage
Source: eLife. 2016 Aug 23;5:e17548. doi: 10.7554/eLife.17548 (PMC5030092; doi:10.7554/eLife.17548)
Supplement: Supplementary file 1. — DOI: http://dx.doi.org/10.7554/eLife.17548.029 [file elife-17548-supp1.docx]

| Primers used in qPCR | Sequence 5’ to 3’ |
| --- | --- |
| GREB1_1 Fwd | AGGCCGCGTGAGTAAAGAG |
| GREB1_1 Rev | TCTGGAGCCAGGAAGACTTG |
| GREB1_2_Fwd | TGCATTACCTCCTATGGCAAG |
| GREB1_2_Rev | CCACTCAATCTGGGGAGATG |
| SLC7A5_1 Fwd | CCCCCACAACTTGTAAACCA |
| SLC7A5_1 Rev | CGAGGGGAGAGGGGTAAG |
| SLC7A5_2 Fwd | GTAGTAAGCAAGGGCCAGGA |
| SLC7A5_2 Rev | CCCCAAAGCCAGTGAGATAC |
| TFF1 Fwd | GAAACTGTACCTAATAACGGCAAAT |
| TFF1 Rev | CATCTGCGACTTGCACTTTG |
| SRF Fwd | TCATTTTGGGGTGTAGACGAT |
| SRF Rev | AAGGATCCAGCTCCTTGTCTC |
| RPL13A Fwd | AGGTGCCTTGCTCACAGAGT |
| RPL13A Rev | GGTTGCATTGCCCTCATTAC |
| MLKL Fwd | TGTAGCTGGGAAGAGCTGGT |
| MLKL Rev | TGCACTTAAACCCCATTGGT |
| 83/84 Fwd | GAACGTTCAGCCTCGTTCTC |
| 83/84 Rev | GGAAGGTGGAAGGAAACACA |

**Supplementary File - Table 1.** Primers used for DRIP-qPCR analysis.
